# Identification of Ethylene Response Factors in Wheat Reveals That *TaERF16-B* Contributes to Salt Tolerance

**DOI:** 10.3390/plants14040621

**Published:** 2025-02-18

**Authors:** Lei Zhang, Aili Wei, Jiating Chen, Lijuan Wu, Tian Li, Linyi Qiao

**Affiliations:** 1 College of Biological Sciences and Technology, Taiyuan Normal University, Taiyuan 030619, China; lei.zhang@tynu.edu.cn (L.Z.);; 2 Shanxi Key Laboratory of Crop Genetics and Molecular Improvement, College of Agronomy, Shanxi Agricultural University, Taiyuan 030031, China; 3 Institute of Crop Sciences, Chinese Academy of Agricultural Sciences, Beijing 100081, China; 4 Ministry of Education Key Laboratory of Molecular and Cellular Biology, Hebei Research Center of the Basic Discipline of Cell Biology, Hebei Key Laboratory of Molecular and Cellular Biology, Hebei Normal University, Shijiazhuang 050024, China

**Keywords:** wheat, *ERF* genes, salt stress, transcriptome, association analysis

## Abstract

Soil salinization is a major abiotic stressor that significantly reduces wheat yield. Identifying novel salt-tolerance genes and integrating them into wheat breeding programs can enhance wheat productivity in saline soils. Ethylene response factor (ERF) plays an important role in plant response to salt stress, and thus far, four wheat *ERF* genes have been identified to be involved in salt stress response. To systematically identify salt tolerance-related *ERF* genes in wheat, in this study, 213 *ERF* sequences were isolated from the whole genome of common wheat and classified into 54 members based on subgenome homology, named *TaERF1* to *TaERF54*. Transcriptome sequencing results showed different expression patterns of *TaERF* members in leaves after 1, 6, 24, and 48 h of NaCl treatment. Based on association analysis, nine *TaERF* genes were correlated with the leaf salt injury index. Among them, five SNPs of *TaERF16-B* formed two haplotypes: Hap1 and Hap2. RT-qPCR results showed that the expression level of *TaERF16-B* was significantly higher in Hap2-typed germplasms than that in Hap1-typed germplasms after 1 and 6 h of NaCl treatment. A Kompetitive Allele-Specific PCR marker K52 was developed for genotyping *TaERF16-B* haplotypes, which further confirmed the significant correlation between *TaERF16-B* and salt tolerance-related phenotypes in mapping population and wheat germplasms. This study provides new genes and molecular markers for improving salt tolerance in wheat.

## 1. Introduction

Soil salinization is a significant constraint on crop production, affecting over 800 million hectares of arable land worldwide [1]. In the context of escalating food demands and a growing population, improving the salt tolerance of crops and thereby enhancing their yield potential is of great significance. Common wheat (*Triticum aestivum* L., AABBDD), as an important cereal crop, is cultivated globally. Mining novel salt-tolerant genes and developing salt-tolerant cultivars is an optimal way of utilizing salt-affected land.

The APETALA2/Ethylene Responsive Factor (AP2/ERF) superfamily is crucial to plants’ responses to abiotic stress [2,3]. This superfamily is defined by the AP2 domain, which contains three anti-parallel β-sheets and one α-helix forming a three-dimensional structure. The YRG element is thought to promote sequence-specific DNA-binding activity, while the RAYD element contains a highly conserved 18-amino acid core region that forms an amphipathic α-helix and thus mediates interactions with protein or DNA [4]. In Arabidopsis, the AP2/ERF superfamily is subdivided into five families: AP2, ERF, DEHYDRATION-RESPONSIVE ELEMENT BINDING (DREB), RELATED TO ABI3/VP1 (RAV) and Soloist [5]. ERF and DREB are the two main subfamilies, which both consist of only one conserved AP2 domain. However, two characteristic conserved amino acid residues in the β-sheet of the AP2 domain distinguish the ERF subfamily from the DREB subfamily. The ERF possesses Ala_14_ and the Asp_19_ [3], which promotes the specific recognition of and binding to the GCC-box element, while the DREB possesses Val_14_ and the Glu_19_ (or occasionally other residues) that are involved in binding to a DRE element [3,6]. In addition, studies have shown that the Ala_37_ of the α-helix, which is invariant in all the ERF and DREB subfamilies, has been demonstrated to be essential for binding to the GCC-box and DRE cis-elements [7].

Among the AP2/ERF superfamily, members of the ERF subfamily are closely related to crop salt tolerance [8,9,10]. At present, several salt stress-responsive *ERF* genes have been identified in various crops, including *ZmERF1* [11] in maize (*Zea mays* L.), *OsERF106* [12] and *OsERF922* [13] in rice (*Oryza sativa* L.), *GmERF75* [14], *GmERF105* [15] and *GmERF135* [16] in soybean (*Glycine max* L.), and *GhERF13.12* [17] in cotton (*Gossypium hirsutum* L.). In common wheat, four salt tolerance-related *ERF* genes have been reported: *TaERF1* was upregulated in response to salt stress, and overexpressing this gene enhanced salt tolerance in Arabidopsis [18]. Overexpression of *TaERF3* resulted in enhanced salt tolerance in wheat plants, while plants with virus-induced gene silencing showed salt sensitivity [19]. *TaERF4* [20] and *TaERF-6-3A* [21], however, are salt-sensitive genes, meaning that plants become more sensitive to salt stress when these genes are overexpressed. Furthermore, a salt-responsive *ERF* gene, *TdERF1*, has been reported in durum wheat (*T. turgidum* L. var. *durum*, AABB). *TdERF1* has four allelic variants, with one variant showing a specific expression pattern in salt-tolerant durum wheat germplasms [22]. However, the wheat ERF family has a large number of members, and it is not yet known whether other *ERF* genes are related to salt tolerance. In this study, we isolated the ERF family from the whole genome of common wheat. Through transcriptome sequencing and association analysis, one salt stress-related gene, *TaERF16-B*, was identified, and a Kompetitive Allele-Specific PCR (KASP) marker for its favorable haplotype was developed for molecular breeding.

## 2. Results

### 2.1. Transcriptional Response to Salt Stress of TaERFs

**Figure 1:**
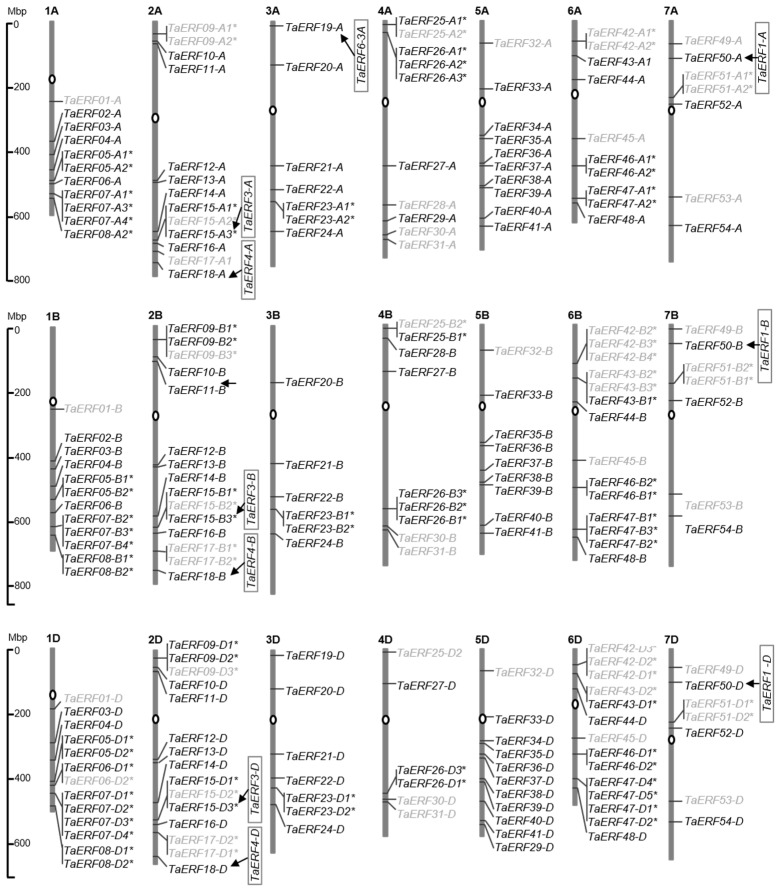
Distribution of *TaERF* genes on wheat chromosomes. Gray genes are not expressed in the transcriptome data of this study. The tandem duplicated genes are marked with asterisks, and the reported names of several *TaERFs* are listed in the boxes. Each chromosome is labeled with number from 1A to 7D above.

**Figure 2:**
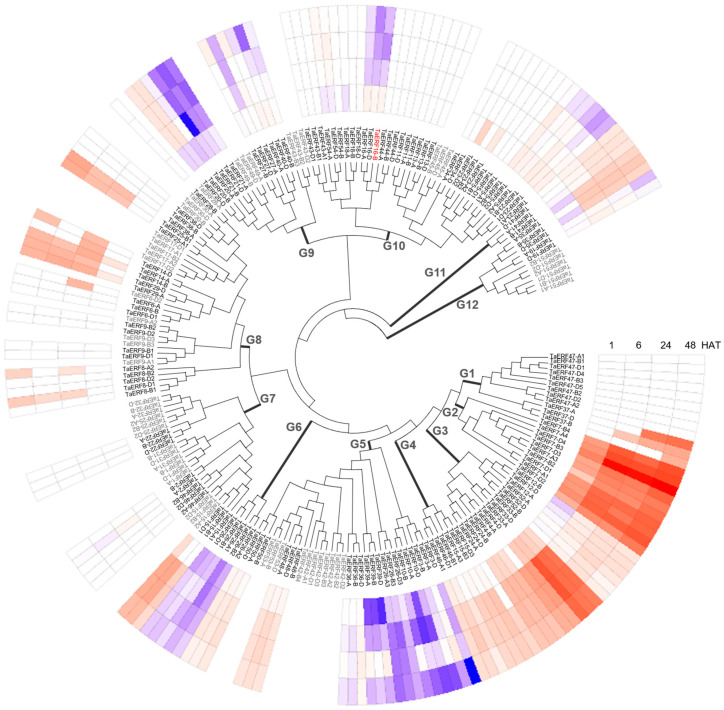
Transcript level of *TaERFs* in salt-tolerant wheat line CH7034 leaves exposed to NaCl stress for 1, 6, 24, and 48 h. FPKM values after salt treatment were subtracted from the value before treatment (0 h) to present different degrees of changes, with upregulation changes appearing in red and downregulation changes appearing in blue; and the gray genes with missing transcriptional data indicate that they were not expressed during the sampling period. *TaERF16-B* is marked in red. HAT: hours after treatment.

A total of 213 sequences containing the AP2/ERF domain, with Ala_14_ and the Asp_19_, were retrieved from the whole-protein sequences of a common wheat, ‘Chinese Spring’, and were distributed across all 21 chromosomes of wheat (Table S1). These were classified into 54 members based on subgenome homology and sequentially numbered from *TaERF1* to *TaERF54* according to their physical position on chromosomes 1~7 (Figure 1 and Figure 2). Among them, 15 *TaERF* members (27.8%) underwent tandem duplication events and were found in gene clusters in the genome, with each gene cluster containing 2~4 tandem duplicated genes (Figure 1). Sequence analysis revealed that *TaERF15.3*, *TaERF18*, *TaERF19-A*, and *TaERF50* are related to the previously reported salt stress-related genes *TaERF3* [19], *TaERF4* [20], *TaERF-6-3A* [21], and *TaERF1* [18], respectively (Figure 1).

Based on multiple sequence alignment, the *TaERF* family was divided into 12 groups (G1~G12, Figure 2). Transcriptome sequencing of leaves in CH7034 revealed that among the 54 *TaERF* members, 10 members (18.5%) detected no expression, 19 members (35.2%) showed no significant change in transcript level after NaCl treatment, 13 members (24.1%) exhibited a sustained upregulation trend under salt stress, 5 members (9.3%) showed a successive downregulation trend, and the remaining 7 members (12.9%) exhibited different degrees of response to salt stress (Figure 2). Overall, *ERF* genes that respond to salt stress were found in each chromosome. In addition, some gene clusters were entirely unexpressed (*TaERF17*, *42*, and *51*), some were fully expressed (*TaERF5*, *7*, *8*, *23*, *26*, *46*, and *47*), and some were partially expressed (Figure 1).

### 2.2. Association Analysis Between TaERFs and Leaf Salt Injury Index

**Figure 3:**
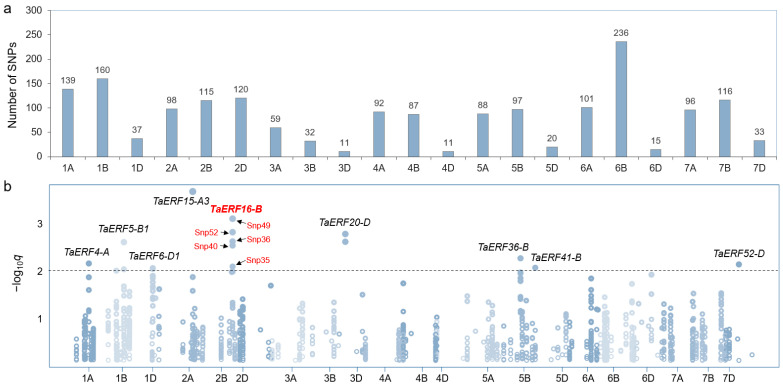
Association analysis between *TaERF* genes and salt-tolerance phenotype of wheat leaves. (**a**) Chromosome distribution of SNPs derived from *TaERF* genes. (**b**) Manhattan plot of association analysis between SNPs and leaf salt injury index of 114 wheat germplasms. The threshold is set to −log_10_*q* > 2. *q* is the *p* value corrected by the FDR. SNPs of *TaERF16-B* are marked in red.

The sequences of *TaERF* family members were blasted against the WheatUnion database, and a total of 1763 Single-nucleotide polymorphisms (SNPs) were identified across all 21 chromosomes of wheat in 117 wheat germplasms (Table S2) (Figure 3a). An association analysis between these 1763 SNPs and the leaf salt injury index (LSI) in the 117 wheat germplasms revealed that 16 SNPs from nine genes were significantly associated with salt tolerance (−log_10_*p* > 2), including *TaERF4-A*, *TaERF5-B1*, *TaERF6-D1*, *TaERF15-A3*, *TaERF16-B*, *TaERF20-D*, *TaERF36-B*, *TaERF41-B*, and *TaERF52-D* (Figure 3b, Table 1 and Table S3). Among them, *TaERF16-B* had five SNPs that were significantly correlated with salt tolerance: Snp35 [A/T] (*p* = 0.0083), Snp36 [C/T] (*p* = 0.0023), Snp40 [T/C] (*p* = 0.0028), Snp49 [C/A] (*p* = 0.0007), and Snp52 [C/G] (*p* = 0.0014) (Figure 3b).

### 2.3. Haplotypes of Salt Tolerance-Related TaERF16-B

**Figure 4:**
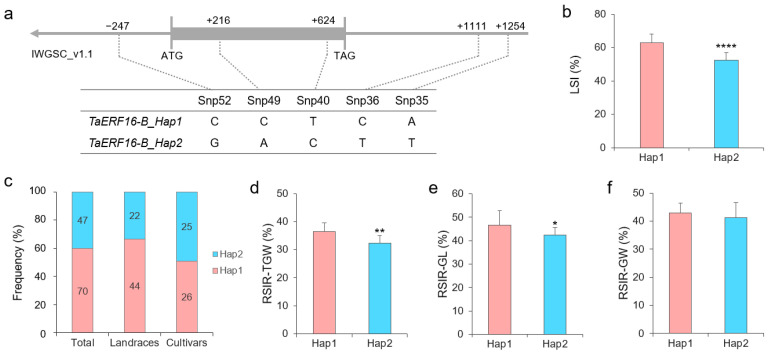
Haplotypes of *TaERF16-B*. (**a**) Location of five SNPs in the gene. Gray box indicates an exon. (**b**) Phenotypic differences in leaf salt injury index (LSI) between Hap1 and Hap2. **** indicates *p* < 0.0001 according to a *t* test. (**c**) Distribution frequency of the two haplotypes in 117 wheat germplasms, including 66 landraces and 51 cultivars. The germplasm numbers are marked in the columns. (**d**) Phenotypic differences in relative salt injury rate (RSIR) of thousand-grain weight (TGW) between Hap1 and Hap2. ** indicates *p* < 0.01 according to a *t* test. (**e**) Phenotypic differences in RSIR of grain length (GL) between Hap1 and Hap2. * indicates *p* < 0.05 according to a *t* test. (**f**) Phenotypic differences in RSIR of grain width (GW) between Hap1 and Hap2.

There are no introns in *TaERF16-B*. Snp52 is located at −247 bp in the promoter region of *TaERF16-B*, while Snp49 and Snp40 are located at positions 216 bp and 624 bp in the coding region of *TaERF16-B*, respectively, and are synonymous mutations. Snp36 and Snp35 are located at positions 1111 bp and 1254 bp in the 3′-UTR of *TaERF16-B*, respectively (Figure 4a). These five SNPs from *TaERF16-B* displayed two haplotypes, Hap1 and Hap2, in the 117 wheat germplasms used in this study. Hap2 had a lower LSI (*p* = 7.18 × 10^−5^) than Hap1, indicating enhanced salt tolerance (Figure 4b). In all 117 wheat germplasms, the distribution frequency of Hap1 and Hap2 was 59.83% and 40.17%, respectively. Hap2 was found in 33.33% of landraces and increased to 49.02% in cultivated varieties (Figure 4c). Moreover, *TaERF16-B* was associated with the relative salt injury rate (RSIR) of grain phenotypes of wheat germplasms. Compared with the Hap1, Hap2 decreased the RSIR of thousand-grain weight (*p* = 0.006, Figure 4d) and grain length (*p* = 0.02, Figure 4e).

### 2.4. Expression Patterns of TaERF16-B_Hap1 and _Hap2

**Figure 5:**
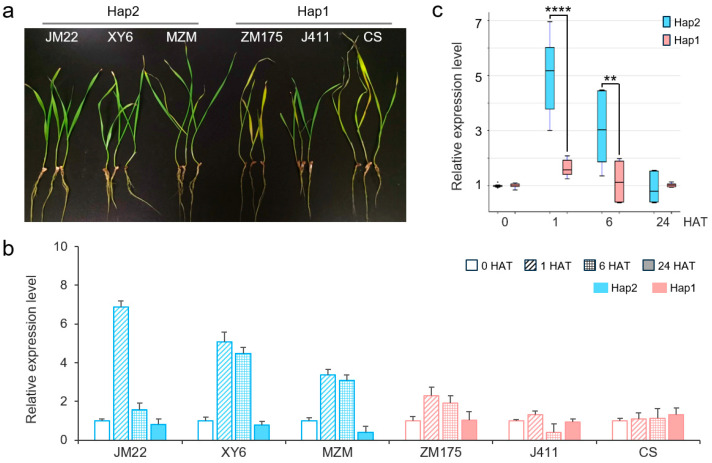
Expression patterns of *TaERF16-B* in Hap1- and Hap2-type wheat germplasms. (**a**) Plant phenotypes after 10 days of 250 mmol/L NaCl solution stress. (**b**) The expression level of *TaERF16-B* at 0, 1, 6, and 24 HAT in wheat leaves. (**c**) Comparison of expression levels of the two haplotypes of *TaERF16-B* in Hap1- and Hap2-type wheat germplasms. ** indicates *p* < 0.01, and **** indicates *p* < 0.0001, according to a *t* test.

According to the transcriptome data in this study, the expression level of *TaERF16-B* was significantly upregulated after 1 h of salt stress and then continued to decrease (Figure 2). To analyze the expression patterns of *TaERF16-B* haplotypes, three germplasms containing Hap2, including cultivar (cv.) Jimai22, cv. Xiaoyan6, and landrace Mazhamai, and three germplasms containing Hap1, including cv. Zhongmai175, cv. Zhongjing411, and landrace Chinese Spring, were randomly selected from the 117 germplasms and subjected to salt stress treatment. Compared with germplasms containing Hap1 that exhibited yellow leaves, Hap2-typed germplasms showed lighter salt injury and were more salt-tolerant (Figure 5a). The expression of *TaERF16-B* was measured at 0, 1, 6, and 24 h after treatment (HAT). In the salt-tolerant Hap2 germplasms, *TaERF16-B* was significantly upregulated at 1 HAT before gradually declining, consistent with transcriptome data. However, this trend was not significant in Hap1-typed germplasms (Figure 5b). Overall, at 1 and 6 HAT, the expression level of *TaERF16-B* in Hap2 germplasms was significantly higher than that in Hap1 germplasms (Figure 5c).

### 2.5. Confirmation of the Salt Tolerance of TaERF16-B in an RIL Population

**Figure 6:**
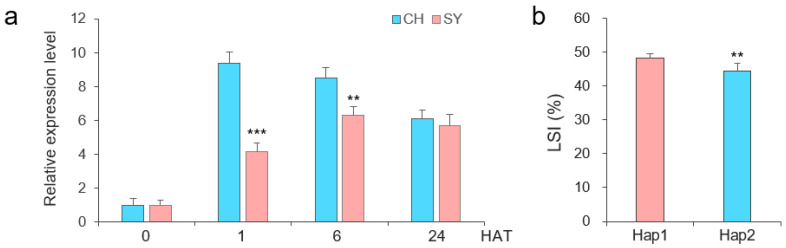
*TaERF16-B* in CH7034 × SY95-71 RILs population. (**a**) The expression level of *TaERF16-B* at 0, 1, 6, and 24 HAT in leaves of CH7034 and SY95-71. (**b**) Phenotypic differences in LSI between Hap1 and Hap2 in RILs population, genotyped by KASP marker K52. ** indicates *p* < 0.01, and *** indicates *p* < 0.001, according to a *t* test.

A recombinant inbred line (RIL) population was constructed using the salt-tolerant breeding line CH7034 and the salt-sensitive breeding line SY95-71. Sequencing results showed that CH7034 carried *TaERF16-B_Hap2*, while SY95-71 carried *TaERF16-B_Hap1* (Table S4). Expression analysis showed that the expression level of *TaERF16-B_Hap2* in CH7034 was significantly higher than that in SY95-71 at 1 and 6 HAT (Figure 6a), which was consistent with the results from the previous experiment (Figure 5c), indicating that differences in the expression levels of different haplotypes may affect plant salt tolerance, and the transcription process of *TaERF16-B* may be regulated by upstream factors. We transformed Snp52, located in the promoter region of *TaERF16-B*, into a KASP marker K52 which was used to type the CH7034 × SY95-71 RIL population lines. K52 accurately distinguished the haplotype of *TaERF16-B* in each line, showing an excellent genotyping efficiency (Figure S2). Association analysis showed that K52 was significantly correlated with the LSI (Figure 6b).

### 2.6. Co-Expression Network of TaERF16-B

**Figure 7:**
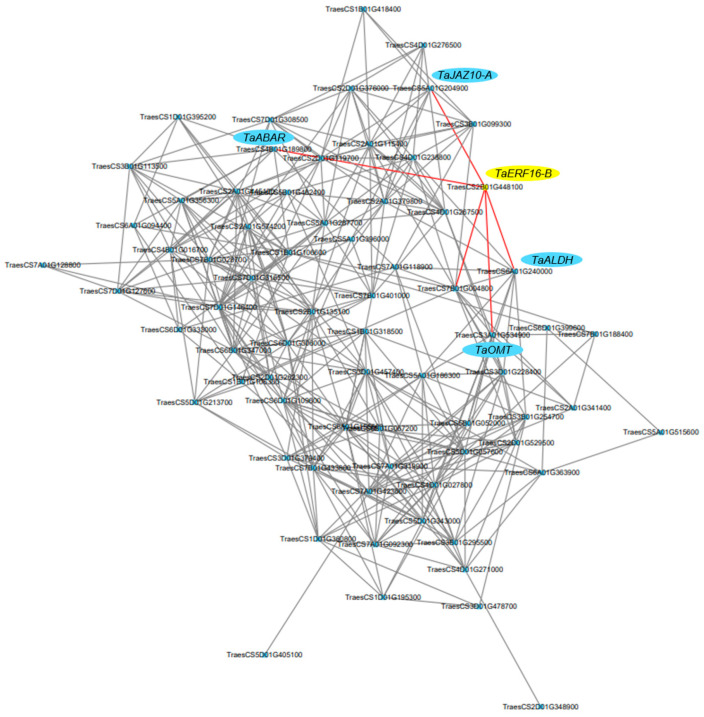
Co-expression network analysis results for *TaERF16-B* and 5911 DEGs containing GCC-box elements in the promoter region. The red lines indicate the possible interactions between *TaERF16-B* and DEGs.

As a transcription factor, TaERF16-B may also be involved in regulating the expression levels of downstream target genes. A total of 5911 differentially expressed genes (DEGs) containing GCC-box elements in their promoter regions were obtained from the transcriptome data of CH7034 leaves under salt stress. A co-expression network between *TaERF16-B* and these DEGs was constructed, and the results showed that five genes had expression patterns that were consistent with *TaERF16-B* (Figure 7). Among them, *TraesCS7B01G004800* was excluded due to its low expression level (FPKM < 1). *TraesCS5A01G204900*, designated as *TaJAZ10-A* [23], which encodes Jasmonate zim-domain protein (TaJAZ), is a transcriptional repressor that regulates the expression of downstream genes in the JA signaling pathway. *TraesCS4B01G189800* encodes Abscisic acid (ABA)-receptor (TaABAR), a receptor for ABA that transduces signals in the ABA pathway. *TraesCS3A01G534900* encodes O-methyltransferase (TaOMT), which is involved in melatonin biosynthesis, and *TraesCS6A01G240000* encodes Aldehyde dehydrogenase (TaALDH) that responds to stress by participating in the metabolism of proline, γ-aminobutyric acid, and lysine.

**Figure 8:**
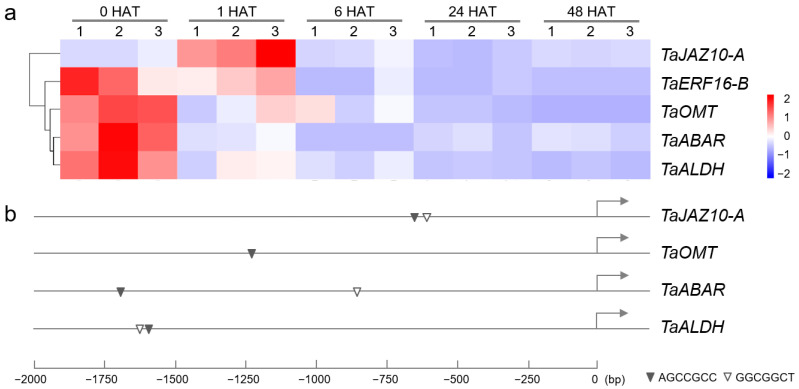
Predicted target genes of *TaERF16-B*. (**a**) Cluster diagram of the transcription data of *TaERF16-B* and four DEGs under NaCl treatment. FPKM values were normalized by z-score to present different degrees of upregulation, with small upregulation changes appearing in blue and large upregulation changes appearing in red. (**b**) GCC-box in the promoters of putative target genes. The black and white triangles respectively represent the forward and reverse complementary conserved sequences of GCC-box, and the arrows represent the transcription direction.

Among the above-mentioned genes, *TaJAZ10-A* and *TaERF16-B* were both significantly upregulated at 1 HAT, with the highest consistency in their expression levels. There is one GCC-box element each at −608 bp and −646 bp in the promoter region of *TaJAZ10-A*. the *TaCOMT* promoter region contains one GCC-box element at −1240 bp; the *TaABAR* promoter region contains GCC-box elements at −804 bp and −1685 bp; and the *TaALDH* promoter region contains GCC-box elements at −1652 bp and −1671 bp (Figure 8).

## 3. Discussion

The response of plant cells to salt stress can be roughly divided into four phases: the early signal transduction phase (within 5 min), the stop phase (from 5 min to 5 h), the quiescent phase (from 5 to 9 h), and the growth recovery phase (from 9 h onwards) [24]. Starting in the stop phase, hormone signaling pathways such as ET, ABA, and JA are activated to regulate downstream gene expression. On the one hand, large amounts of compounds such as proline, alkaloids, etc., are synthesized to increase cytoplasmic concentration and alleviate osmotic stress. On the other hand, superoxide clearance mechanisms are increased to alleviate oxidative stress [24]. The ERF family plays an important role in regulating plant salt tolerance through the ET signaling pathway [8].

### 3.1. Response of Wheat ERF Family Genes to Salt Stress

In this study, a total of 54 ERF members, which were distributed across all 21 chromosomes, were isolated from the whole genome of common wheat. Among them, 15 *TaERFs* experienced tandem duplication events and were present in clusters. Clustered *ERF* genes that have overlapping or unique regulatory functions, as well as the ability to regulate each other mutually, are particularly advantageous for balancing the regulation of metabolic pathways involved in defense and growth [25]. In the model crop, tobacco, plants defend biotic stress by synthesizing large amounts of alkaloids (mainly nicotine). The two well-known genetic loci, *NICOTINE1* (*NIC1*) and *NIC2*, on the tobacco chromosome involved in nicotine synthesis are both *ERF* gene clusters [26]. Interestingly, these two *ERF* clusters were strongly induced by salt stress [27]. In this study, genes within seven *ERF* clusters, namely *TaERF5*, *7*, *8*, *23*, *26*, *46*, and *47*, were all expressed, and these loci may be involved in the metabolism of certain compounds to enhance tolerance to salt stress.

Within the *TaERF* family, *TaERF15.3* (previously reported as *TaERF3*), *TaERF18* (previously reported as *TaERF4*), *TaERF19-A* (previously reported as *TaERF-6-3A*), and *TaERF50* (previously reported as *TaERF1*) have been confirmed to be involved in the salt stress response in wheat [18,19,20,21]. Clustering analysis of the *TaERF* family with known salt-tolerant genes in plants (Figure S1) showed that *TaERF12* and the salt-tolerant gene *JcERF2* identified in *Jatropha curcas* (a drought- and salt-tolerant oil plant) [28] were located on the same branch, *TaERF22,* and the rice salt-sensitive gene *OsERF922* [13] was also on the same branch, and *TaERF40* and the tobacco salt-tolerant gene *Tsi1* [29] were on the same branch, indicating that these three *TaERF*s may also exhibit similar functions in terms of their salt stress response.

### 3.2. TaERF16-B Is Related to Salt Tolerance

In this study, seven genes of the *TaERF* family involved in salt stress response and associated with the salt-tolerant phenotype in leaves were screened, namely *TaERF4-A*, *TaERF15-A3*, *TaERF16-B*, *TaERF20-D*, *TaERF36-B*, *TaERF41-B*, and *TaERF52-D*. Apart from *TaERF15-A3*, which has been reported previously [19], the remaining six genes were novel. Among them, *TaERF16-B* constituted two haplotypes in 117 wheat germplasms, and the Hap2-typed germplasms showed better salt tolerance. The frequency of Hap2 in cultivated wheat was 15.69% higher than that in landrace wheat, indicating that the *TaERF16-B* locus may have undergone artificial selection. The expression level of *TaERF16-B_Hap2* during the stop phase (1 and 6 HAT) under salt stress was significantly higher than that of *TaERF16-B*_Hap1, suggesting the transcription process of *TaERF16-B* might be regulated by upstream factors. Snp52 was located in the target site of the histone acetyltransferase P300 in the promoter region of *TaERF16-B* [30]. A wheat histone acetyltransferase, TaHAG1, has been confirmed as a key regulatory factor in enhancing wheat salt tolerance [31], and it is hypothesized that the single base mutation of Snp52 might have affected the regulatory effect of upstream factors. One marker, K52, was developed on Snp52, which was shown to be significantly associated with LSI in the CH7034 × SY95-71 RIL population, and could be further used for wheat germplasm screening or marker-assisted breeding. Furthermore, several ABRE elements were found at the −494 bp, −596 bp, −1114 bp, −1324 bp, −1331 bp, and −1584 bp in the promoter region of *TaERF16-B*. It is known that wheat ABRE-BINDING FACTOR2 (TaABF2) can regulate the transcription level of *TaERF87* by binding to its ABRE elements [32], and further validation will be achieved in our future studies. In addition, *TaERF16* showed a close genetic relationship and high sequence similarity with *SodERF3* in sugarcane (*Saccharum officinarum* L.) (Figure S1). *SodERF3* encoded a 201-amino acid DNA-binding protein which can bind to the GCC box. Northern and Western blot analysis showed that *SodERF3* is induced by ethylene, ABA, and salt stress. Overexpression of *SodERF3* in tobacco plants enhanced salt and drought tolerance [33], indicating that *TaERF16-B* may also exhibit similar functions in the salt stress response.

### 3.3. Predicted Target Genes of TaERF16-B Protein

In this study, four genes, namely *TaJAZ10-A*, *TaABAR*, *TaOMT*, and *TaALDH*, that contained GCC-box elements in their promoter regions and exhibited similar expression patterns to *TaERF16-B*, were identified. When crops respond to salt stress, the ethylene signaling pathway will crosstalk with other hormone signaling pathways through ERF transcription factors [34,35].

The predicted target genes, *TaJAZ10-A* and *TaABAR*, belong to the JA and ABA signaling pathways, respectively. Under salt stress, *TaJAZ10-A* was shown to be upregulated at 2 and 4 HAT and then decreased in our previous study [23], which was consistent with the trend observed in this study. Some JAZs in crops, such as *OsJAZ8* [36] and *OsJAZ9* [37] in rice, *TdTIFY11a* [38] in durum wheat, *TaTIFY3B* and *TaTIFY10A* in common wheat [39], have been demonstrated to regulate salt tolerance. Therefore, *TaJAZ10-A* may be a target gene of TaERF16-B. In addition, studies on the protein interaction patterns between ERF and JAZ have been reported [40,41], and further studies will be conducted to explore the interaction between TaERF16-B and TaJAZ10-A. Another predicted target gene, *TaABAR*, belongs to the ABA signaling pathway. In cotton, the *ABAR* genes *PYRABACTIN RESISTANCE1-3A* (*GhPYR1-3A*) and *PYR-LIKE9-5D* (*GhPYL9-5D*) were induced by salt stress [42], and overexpression of *OsPYL5* in rice improved plant salt tolerance [43]. To date, no studies on salt-related *ABAR* genes in wheat have been reported. However, it was found that *TaABAR* in this study was the B-genome copy of the known wheat drought-resistant gene *TaPYL4* [44]—that is, *TaPYL4-4B*—which indicates that *TaPYL4* might be involved in the response to various abiotic stresses.

The other two predicted target genes encode enzymes. It has been reported that the protein TaALDH can act as a proline catabolism enzyme, and the *ALDH* gene was downregulated under salt stress to allow the accumulation of proline as a compatible solute [45]. *ZmALDH22A1* in maize [46] and *AhALDH3H1* in peanut (*Arachis hypogaea* L.) [47] were also involved in this regulatory process. However, only one *TraeALDH7B1-5A* in wheat has been reported to be involved in drought resistance [48], and no study has investigated salt tolerance yet. *TaOMT* encodes O-methyltransferase. It has been reported that an *OMT1* gene located on the long arm of chromosome 7B in wheat is a hub gene involved in the response network to both drought and salt stress, which enhances the antioxidant system and reconstructs the redox state by regulating melatonin biosynthesis, and promotes wheat’s tolerance to drought and salinity stress [49].

## 4. Materials and Methods

### 4.1. Plant Materials

Transcriptome sequencing was conducted on the salt-tolerant wheat breeding line CH7034 under salt stress. A recombinant inbred line (RIL) population of 184 lines, derived from a cross between CH7034 and the salt-sensitive line SY95-71 [50], along with 117 wheat germplasm accessions [51], was used for association analysis. The set of 117 wheat germplasm is part of the Chinese mini core collection, which includes 66 landraces and 51 modern cultivars. CH7034, SY95-71, and six other wheat germplasms, including Jimai22, Xiaoyan 6, Zhongmai175, Jing411, Mazhamai, and Chinese spring, were used for RT-qPCR analysis.

### 4.2. Isolation of ERF Sequences from the Wheat Genome

Hidden Markov Model files of the AP2/ERF superfamily (PF00847.20, http://pfam.xfam.org/, accessed on 10 March 2024) were employed to retrieve annotated protein sequences of Chinese Spring (version 1.1, http://wheat-urgi.versailles.inra.fr/, accessed on 10 March 2024) via HMMER software (version 3.0, HHMI Janelia Farm Research Campus, Ashburn, VA, USA). Sequences containing the AP2 domain were obtained, and those which belonged to the ERF subfamily, Ala_14_ and Asp_19_ in the AP2 domain, were further screened as wheat ERF sequences. The number of members was determined based on the homologue of A/B/D subgenomes (http://plants.ensembl.org/Triticum_aestivum/Info/Index, accessed on 10 March 2024), and genes within 10 Mb were defined as tandem duplicated genes and numbered according to their linear arrangement on the chromosome. A phylogenetic tree was constructed using MEGA (version 6.0, Arizona State University, Tempe, AZ, USA).

### 4.3. Transcriptional Data of TaERF Genes After Salt Treatment

The transcriptional data used in this study were from our previous research [52]. Briefly, three-leaf-stage seedlings of CH7034 were subjected to salt stress treatment with 250 mmol/L NaCl, and the leaves were harvested at 0, 1, 6, 24, and 48 h, with three biological replicates for each sample. Transcriptome sequencing was performed on the Illumina platform (Biomarker Tech., Beijing, China). The clean reads were mapped to the Chinese Spring reference genome (version 1.0, http://wheat-urgi.versailles.inra.fr/, accessed on 20 May 2024) to obtain the assembled gene sequences. The wheat ERF sequences obtained previously were then compared with these assembled genes to obtain their expression data under salt treatment at different time points, which were measured by fragments per kilobase of exon model per million mapped fragments (FPKMs) values calculated from the following formula:

FPKM = cDNA Fragments/Mapped Fragments (Millions) × Transcript Length (kb)(1)

where “cDNA Fragments” represents the number of fragments mapped to a certain transcript, “Mapped Fragments (Millions)” represents the total number of fragments mapped to the transcript, measured in units of 10^6^, and “Transcript Length (kb)” represents the length of a certain transcript, measured in units of 10^3^ bases. Differential expression analysis between the control group (0 h of NaCl treatment) and the salt stress group (1, 6, 24, and 48 h of NaCl treatment) was performed using the DESeq package (version 2), with a threshold of |log_2_FoldChange| ≥ 1 and a False Discovery Rate (FDR) of ≤0.01.

### 4.4. Association Analysis of TaERF Genes

Three-leaf-stage seedlings of 117 wheat germplasms were subjected to salt stress with 250 mmol/L NaCl for 10 days, and the phenotypes of the leaves were investigated. The phenotypes were categorized into five levels: level 0 (normal leaf growth without any symptoms of damage); level 1 (basic normal leaf growth with 2–3 green leaves, while leaf tips turn yellow or dry); level 2 (inhibited leaf growth with only 2 green leaves or 2 leaves with yellow parts); level 3 (significant damage to the leaf growth with only 1 green leaf or very few parts of the leaves remaining green among the 3 leaves); and level 4 (complete or near-death of the leaf). Then, the leaf salt injury index (LSI) of each sample was calculated using the following formula:

LSI (%) = ∑ (0 × *n*_0_ + 1 × *n*_1_ + 2 × *n*_2_ + 3 × *n*_3_ + 4 × *n*_4_) × 100/(4 × *N*) (2)

where *n*_0_ to *n*_4_ represent the number of plants in five different levels, respectively, and *N* is the total number of plants. The experiment was repeated three times, and the average value was taken.

The *TaERF* family sequences were submitted to the WheatUnion database (http://wheat.cau.edu.cn/WheatUnion/, accessed on 7 July 2024), and SNPs in the coding regions and 2000 bp upstream from the start codon of the genes were retrieved and analyzed to determine their association with the leaf salt injury index in the 117 wheat germplasms using the GAPIT program (version 3, Washington State University, Pullman, WA, USA) in R (version 4.3.1, The University of Auckland, Auckland, New Zealand). The significance threshold was set at −log_10_*q* > 2 (i.e., *q* < 0.01), where *q* is the *p* value corrected by the FDR.

### 4.5. Association Analysis of TaERF16-B

The leaf salt injury index of CH7034 × SY95-71 RIL population lines was calculated using the method described in Section 4.4. Moreover, the set of wheat germplasms with 117 accessions was planted in two experimental fields at Dongyang Experimental Demonstration Base of Shanxi Agricultural University (E112°40′, N37°33′, Jinzhong, China) in early November of 2023. The underground of the two experimental fields was isolated with concrete to prevent salt being exchanged with the surrounding soil, and the soil salinity was controlled at 0 (CK) and 0.3% (m/m), respectively. Briefly, fifteen seeds from each germplasm were planted in a row of 1.5 m in length, with spacing of approximately 10 cm. After harvesting, 10 plants were randomly selected from each material and their grain phenotypes, such as their thousand grain weight, were measured using instruments. After harvesting in 2024, ten plants from each germplasm were randomly selected to measure their grain phenotypes, including their thousand-grain weight (TGW), grain length (GL), and grain width (GW), using the TPKZ-3 intelligent seed test and analysis system (Top Cloud-Agri Technology Co., Ltd., Hangzhou, China), and then the average value was calculated. Finally, the relative salt injury rate (RSIR) of each grain phenotype was calculated using the following formula:

RSIR (%) = (*X*_CK_ − *X*_NaCl_)/*X*_CK_ × 100% (3)

A KASP marker was developed on Snp52 detected in the promoter region of *TaERF16-B* (K52-F1: 5′-TTGCGCACCAGTACACACC-3′; K52-R1: 5′-GAAGGTGACCAAGTTCATGCTgttcctccgctaatacctctC-3′; K52-R2: 5′-GAAGGTCGGAGTCAACGGATTgttcctccgctaatacctctG-3′) and used to genotype the RIL population of CH7034 × SY95-71 and the set of wheat germplasms. The association between different genotypes of Snp52 and the salt injury-related phenotypes was analyzed, and the significance threshold was set at *p* < 0.01. The KASP reactions were conducted using a QuantStudio 3 Real-time PCR System (Applied Biosystems, Carlsbad, CA, USA) according to the following steps: denaturation at 94 °C for 10 min, ten cycles of touchdown PCR (94 °C for 20 s; touchdown at 60 °C initially, decreasing by −0.6 °C per cycle for 60 s), and 40 additional cycles (94 °C for 20 s; 55 °C for 60 s). PCR products were detected in a fluorescence scanner under FAM and HEX channels.

### 4.6. RT-qPCR

Three-leaf-stage seedlings were subjected to salt stress with 250 mmol/L NaCl, and young leaves were collected at 0, 1, 6, and 24 h after treatment. Total RNA was extracted using an RNA extraction kit (Tianmo Bio, Beijing, China) and reverse-transcribed into cDNA using a reverse transcription kit (Takara Bio, Shiga, Japan). RT-qPCR was performed on the QuantStudio 3 Real-time PCR System mentioned above, using *TaERF16-B*-specific primers (qF: 5′-GAACAAGGTGGCGTCCCA-3′; qR: 5′-GAGATCGGTCCATCTCCTAT-3′) and Premix Ex Taq II enzyme (Takara Bio, Shiga, Japan), and wheat *ACTIN* gene (Actin-F: 5′-GGAACTGGCATGGTCAAGGCTG-3′; Actin-R: 5′-CCCATCCCCACCATCACACC-3′) was used as the internal reference gene. Each reaction was repeated three times, and the results were analyzed using the 2^−*ΔCT*^ and fold-change method, wherein the *ΔCT* was calculated using the following formula:

*ΔCT* = *CT* (target gene) − *CT* (reference gene) (4)

### 4.7. Co-Expression Network Construction

A co-expression network was generated for *TaERF16-B* based on the RNA-Seq data in Section 4.3. FPKM values of *TaERF16-B* and DEGs with GCC-box elements in the promoter region were min–max normalized and log2-transformed to perform the Weighted Gene Co-Expression Network Analysis (WGCNA) using R (version 4.3.1) software and the WGCNA package (version 1.72, University of California, Los Angeles, CA, USA) to construct a co-expression module. This module was defined as a cluster of highly interconnected genes with similar expression patterns. The pairs of *TaERF16-B* and interconnected genes were selected and visualized with a heatmap on SRplot platform (www.bioinformatics.com.cn/SRplot, accessed on 24 November 2024). Genes with FPKM values below 1 in all samples were defined as non-expressed genes.

## 5. Conclusions

In this study, 54 members of the *TaERF* family were isolated from the whole genome of common wheat, 25 of which were induced under NaCl treatment in the leaves of the salt-tolerant line CH7034. An association analysis was conducted on 117 wheat germplasms, and nine genes that were significantly correlated with the leaf salt injury index were identified. Among them, *TaERF16-B* exhibited two haplotypes in the tested germplasms, with Hap2-typed germplasms corresponding to better salt tolerance, and its expression level in the leaves of wheat at 1 and 6 HAT was significantly higher in Hap2-typed germplasms than in Hap1-typed germplasms. A KASP marker was developed on an SNP detected in the *TaERF16-B* promoter region, which can be further used for wheat germplasm screening or marker-assisted breeding.

## Figures and Tables

**Table 1:** SNPs that significantly correlated with leaf salt injury index.

*TaERF* Genes	SNP	Chromosome	Position (IWGSC v1.0)	*q* Value
*TaERF04-A*	Snp22[A/C]	1A	465,495,549	0.0071
*TaERF05-B1*	Snp13[C/G]	1B	551,869,884	0.0095
*TaERF05-B1*	Snp14[C/T]	1B	551,869,911	0.0024
*TaERF06-D1*	Snp08[T/C]	1D	421,224,882	0.0089
*TaERF06-D1*	Snp24[C/T]	1D	421,227,176	0.0093
*TaERF15-A3*	Snp36[G/A]	2A	673,328,463	0.0002
*TaERF16-B*	Snp35[A/T]	2B	640,764,124	0.0083
*TaERF16-B*	Snp36[C/T]	2B	640,764,267	0.0023
*TaERF16-B*	Snp40[T/C]	2B	640,764,754	0.0028
*TaERF16-B*	Snp49[C/A]	2B	640,765,162	0.0007
*TaERF16-B*	Snp52[C/G]	2B	640,765,624	0.0014
*TaERF20-D*	Snp02[G/A]	3D	124,865,746	0.0023
*TaERF20-D*	Snp03[T/C]	3D	124,866,660	0.0016
*TaERF36-B*	Snp82[C/G]	5B	387,177,561	0.0054
*TaERF41-B*	Snp03[A/G]	5B	653,804,805	0.0088
*TaERF52-D*	Snp01[A/G]	7D	246,072,894	0.0074

## Data Availability

Data are contained within the article and Supplementary Materials.

## Supplementary Materials

The following supporting information can be downloaded at https://www.mdpi.com/article/10.3390/plants14040621/s1, Figure S1: Phylogenetic analysis of *TaERF* genes and known salt-tolerant genes in plants, Figure S2: Genotyping result of KASP marker K52 in the CH × SY RIL population, Table S1: Gene IDs and chromosome position of TaERF family members, Table S2: 117 wheat germplasms used in association analysis, Table S3: Results of the association analysis between all SNPs and the leaf salt injury index in wheat germplasms, Table S4: Sequences of TaERF16-B in wheat lines CH7034 and SY95-71.
