# Supplementary material for: Identification of Ethylene Response Factors in Wheat Reveals That TaERF16-B Contributes to Salt Tolerance
Source: Plants (Basel). 2025 Feb 18;14(4):621. doi: 10.3390/plants14040621 (PMC11859885; doi:10.3390/plants14040621)
Supplement: Supplementary file 1 [file plants-14-00621-s001.zip › FigureS1 and S2_R1.pdf]

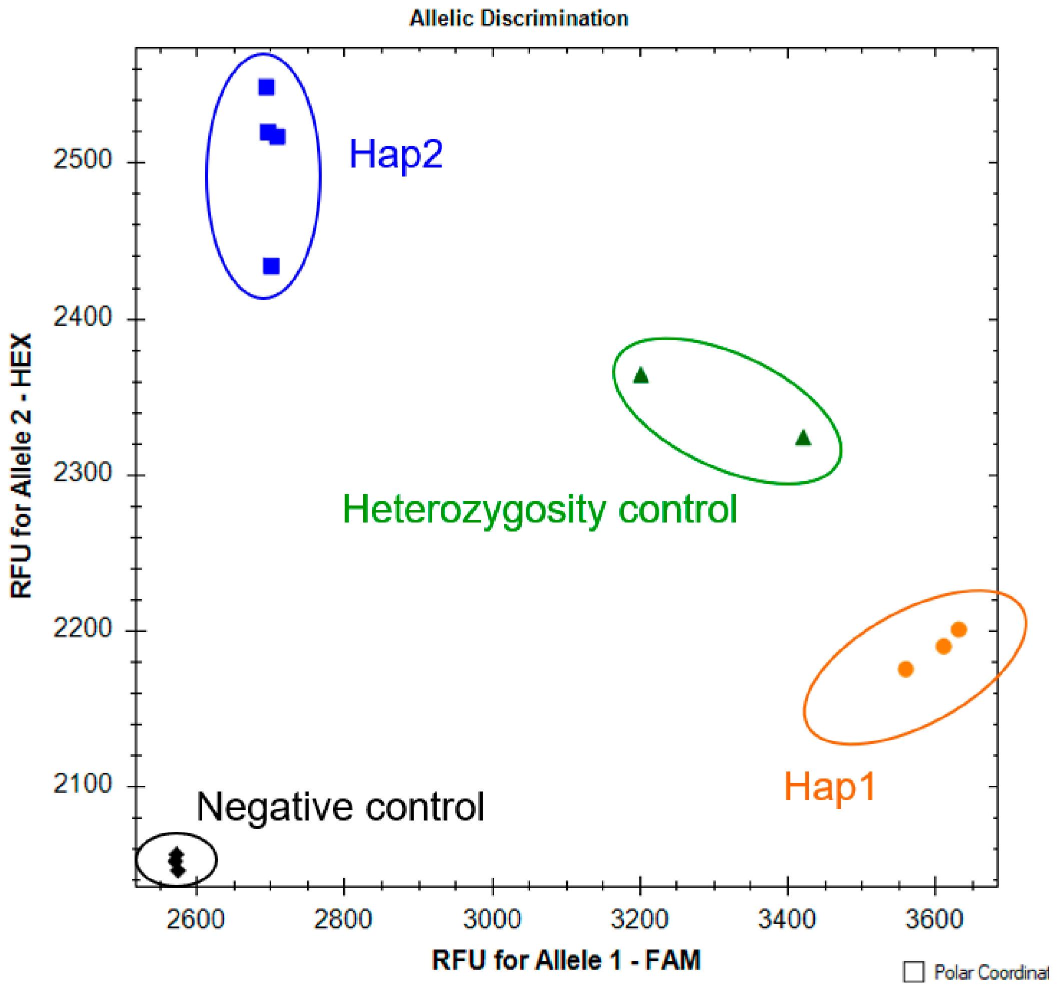

Figure S1. Genotyping result of KASP marker K52 in the CH × SY RIL population. The mixture of equal DNA from parents was used as heterozygous control.

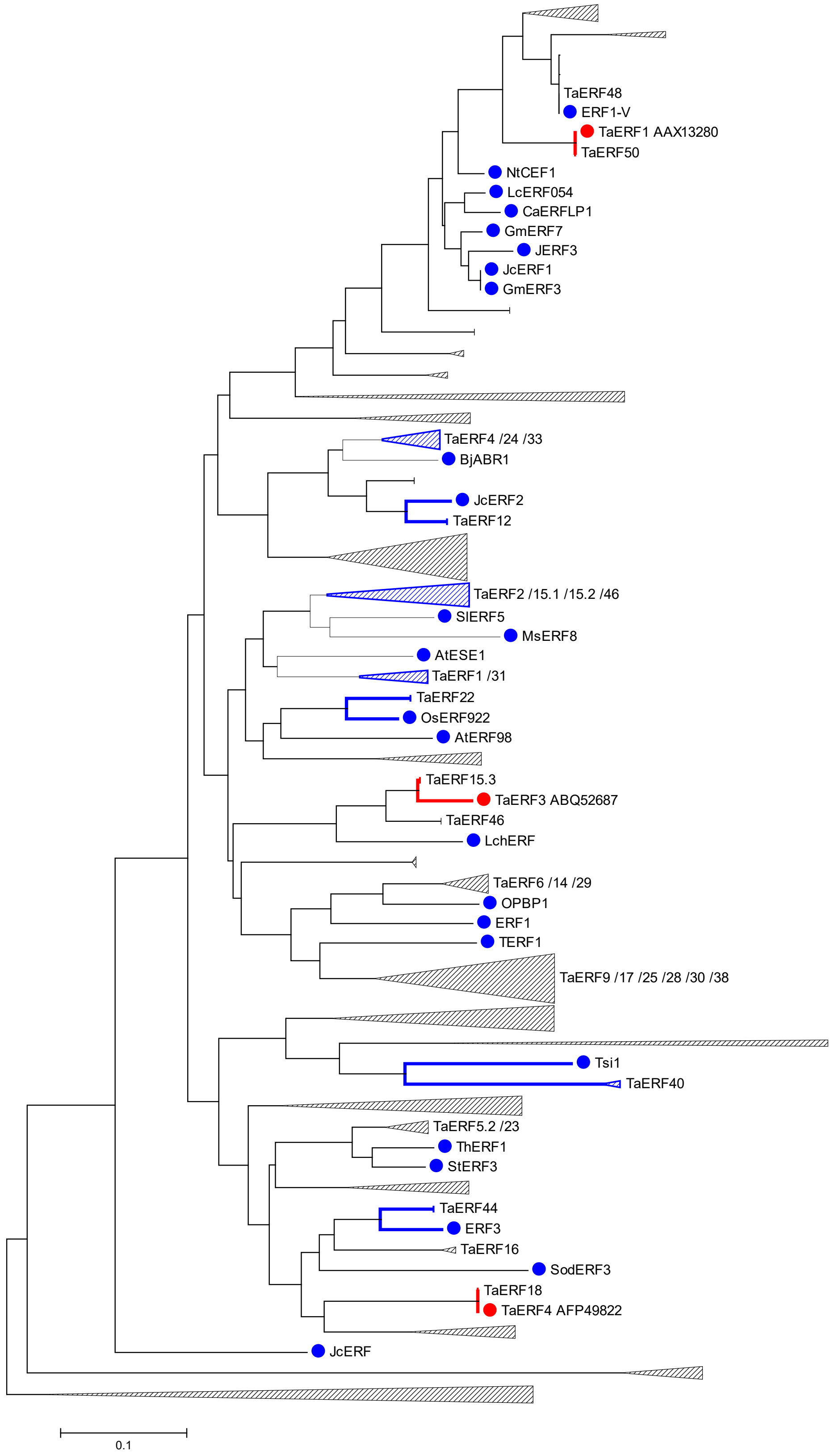

Figure S2. Phylogenetic analysis of *TaERF* genes and known salt tolerant genes in plants.
